# A Case Report of Early Onset Intrahepatic Cholestasis of Pregnancy

**DOI:** 10.7759/cureus.71948

**Published:** 2024-10-20

**Authors:** Olatunji Ogunnubi, Sara Ismail, Rasmus I Okonkwo

**Affiliations:** 1 Obstetrics and Gynaecology, Sherwood Forest Hospitals NHS Foundation Trust, Sutton-in-Ashfield, GBR; 2 Obstetrics and Gynaecology, Doncaster and Bassetlaw Teaching Hospitals NHS Foundation Trust, Doncaster, GBR; 3 Obstetrics and Gynaecology, NHS England, England, GBR; 4 Obstetrics and Gynaecology, NHS Wales, Cardiff, GBR

**Keywords:** bile acid, intrahepatic cholestasis, intrahepatic cholestasis of pregnancy (icp), liver disease, obstetric cholestasis, pruritus, pruritus in pregnancy

## Abstract

Intrahepatic cholestasis of pregnancy (ICP) is a liver disorder occurring in pregnancy presenting with pruritus and the presence of increased levels of serum bile acid. Early onset ICP occurs when this presents before 24 weeks of gestation. This is a rare presentation but can pose a great clinical dilemma. Most often, there are no considerable maternal problems from ICP other than pruritus, which sometimes significantly impacts the quality of life. However, there can be adverse foetal outcomes and complications such as stillbirth.

We present the case of an early onset presentation of ICP in a 27-year-old primigravid woman with pruritus and rapidly rising serum bile acid concentration. This report highlights the clinical course and presentation, diagnostic workup, and management approach. In the second trimester, the patient’s bile acid levels rose rapidly from 15 μmol/L to 273 μmol/L (normal range: 0-18 μmol/L) over a short time. Her alanine aminotransferase also increased from 22 U/L to 141 U/L (normal range: 0-55 U/L). This prompted close feto-maternal monitoring and swift, robust intervention. She later on went into preterm labour at 30 weeks’ gestation and delivered via category 1 lower segment caesarean section due to foetal distress in labour. We highlight through this case that early recognition, close monitoring, and multi-disciplinary care are key to optimise maternal and foetal outcomes in early onset ICP.

## Introduction

Intrahepatic cholestasis of pregnancy (ICP) is a disorder of unknown aetiology. It is the most common pregnancy-associated liver disease, with a variable incidence worldwide between 0.2% and 25% [1,2]. In the United Kingdom, ICP affects 0.7% of pregnancies in the multi-ethnic population [3]. The classical presentation of ICP is pruritus, which cannot be traced to any other pathology. It is a liver disorder in pregnancy that presents with pruritus in the presence of raised levels of bile acids. It typically presents in pregnancy after 30 weeks of gestation [4]; however, a few cases have been diagnosed in the first trimester [5]. Early onset ICP occurs when this presentation manifests before 24 weeks of gestation. Although this is somewhat rare, it creates considerable clinical complexities.

This article was previously presented as an abstract (oral presentation) at the 15th Congress of the European Society of Gynaecology in Amsterdam on December 2, 2023.

## Case presentation

A 27-year-old gravida 1 woman presented at 22 weeks of gestation with pruritus. There were no other symptoms such as rash, and she had no pre-existing conditions. Her serum bile acid was tested, which came back as 15 μmol/L (normal range: 0-18 μmol/L). We ordered a repeat testing five days later, and the serum bile acid level had declined to 7 μmol/L. A liver function test was also conducted, which revealed normal alanine aminotransferase (ALT) of 18 U/L (normal range: 0-55 U/L). Her values remained stable within the normal range for around seven weeks. At 26 weeks’ gestation, she presented again with persistent pruritus, which was worse on her hands, legs, and soles of the feet. Her symptoms were reported as being more prominent at night time, resulting in sleep disruptions and insomnia. On examination, there were no rash or any skin manifestations or signs. There were also no peripheral stigmata of liver disease. No other liver pathology was identified; hence, the diagnosis of ICP was maintained with a bile acid of 25 μmol/L. Her ALT remained normal at 22 U/L. Subsequently, at 29 weeks and 5 days’ gestation, on repeat testing her bile acid levels increased to 273 μmol/L. Also, her ALT increased from 22 U/L to 141 U/L, prompting close feto-maternal monitoring and the need for prompt intervention as the bile acid levels increased quickly, progressing fast in a short time.

At this stage, symptomatic treatment was commenced, and a management plan was made according to recommendations from the Green-top Guideline No. 43 of the Royal College of Obstetricians and Gynaecologists. This included testing and close monitoring of bile acids and other biochemical/liver markers, as well as testing for gestational diabetes mellitus (GDM) as ICP increases the risk of GDM. As her bile acid levels were >100 μmol/L, induction of labour was planned for 35-36 weeks. Additionally, we constantly kept a watch on the presence of risk factors or comorbidities and ensured that it was quickly picked up, if any, as the pregnancy advanced. Her symptoms of pruritus also worsened (more severe at night with insomnia); hence, chlorpheniramine and ursodeoxycholic acid (UDCA) 1,000 mg/day (500 mg twice daily) were commenced. Her management plan included trice weekly (alternate days) foetal surveillance (regular cardiotocography [CTG], Doppler scan with pulsatility index, and growth scan) and biochemical testing, as well as involvement of a hepatologist and neonatologist. A hepatologist reviewed the patient with all liver profile investigations that were conducted, which excluded any primary liver pathology. A neonatologist was also a part of her management and foetal surveillance plan corroborated, in addition to plans made for neonatal care, should the foetus develop any of the known foetal complications.

She also underwent a full non-invasive liver screening, which included liver function tests, hepatitis serology, other viral screening such as TORCH (toxoplasmosis, rubella, cytomegalovirus, herpes simplex, and HIV) and Parvovirus, coagulation screening, and liver ultrasound scan. All results were normal with no other liver pathology identified. Abdominal ultrasound revealed normal liver architecture, with no abnormalities seen. She subsequently went into pre-term labour spontaneously at 30 weeks’ gestation, before the date we had planned her for induction of labour. She delivered a live male neonate in good condition via category 1 lower segment caesarean section due to intrapartum foetal distress. Apgar score at delivery was 7/10 at 1 minute and 9/10 at 5 minutes. Her symptoms resolved after a few days postpartum, and UDCA was discontinued. At three weeks postpartum, her bile acids and liver biochemistry had become normal. Her newborn was admitted to the neonatal care unit due to prematurity and was discharged after three weeks. Other relevant results are shown in Table 1.

**Table 1:** Other relevant results ALT, alanine aminotransferase

Parameters	Delivery day	1 day post-partum	2 days post-partum	3 weeks post-partum	Reference range
Bile acids	253	91	48	5	0-18 μmol/L
ALT	171	144	130	32	0-55 U/L

## Discussion

ICP is a gestation-specific liver disorder, defined most often as the onset of pruritus, usually from the third trimester of pregnancy, and associated with abnormal liver test results and/or increased total serum bile acids and spontaneous relief after delivery [6]. However, when it occurs much earlier, a more active approach is needed for foetal surveillance and close monitoring. Whilst ICP usually has no significant maternal adverse outcome aside from decreased quality of life from pruritus, it can lead to significant foetal complications including stillbirth [7]. Other foetal complications include preterm delivery, low birth weight, cardiac arrhythmias, meconium-staining of amniotic fluid, infant respiratory distress syndrome, and sudden perinatal death [2]. The higher the serum level of bile acids, the more at risk the foetus of developing these complications. It is important to consider ICP as a diagnosis when pregnant women present with concerning and persistent pruritus, which is often intense, mainly on the hands and soles of the feet, in the absence of a rash. The risk factors for ICP are both genetic and environmental [1]. Family clustering and varying incidence in different geographic regions suggest an underlying genetic predisposition [2]. We did not investigate this patient for possible genetic predisposition as she had no background liver condition or any risk factors. Moreover, this was her first pregnancy, hence no previous ICP presentations.

The gold standard for diagnosing ICP is serum bile acid level ≥19 μmol/L. Feto-maternal monitoring is essential. Weekly maternal liver function tests, bile acids, clotting profile, urinalysis, blood pressure monitoring, and testing for GDM are important as ICP increases the risk of preeclampsia and GDM. In terms of foetal monitoring, CTG and Doppler scan should be used when indicated, though they do not predict or prevent stillbirth [8]. Multi-disciplinary care is also required. A hepatologist is needed in severe, very early, and atypical presentation of ICP [8]. Neonatologists should also be part of the team to ensure optimal outcomes.

There are no disease-specific treatments for ICP. Furthermore, no pharmacological treatments have been proven to improve foetal outcomes [7,8]. Symptomatic treatments include topical emollients, and oral anti-histamines are often routinely offered to try to alleviate pruritus; however, their effectiveness tends to be limited [7,8]. In addition, UDCA, a naturally occurring secondary bile acid, is often used to also alleviate the symptoms of ICP. The role of drug treatment in ICP is to try to reduce maternal itching (which may be of variable intensity and is unrelated to bile acid concentrations) [8]. Previously, it was thought that improvements in clinical symptoms and biochemical indices of liver function consistently occur following UDCA administration [9]. However, in a more recent and larger trial, maternal bile acid concentrations were found to be higher in the group treated with UDCA [8,10], possibly as a result of standard laboratory assays being unable to distinguish between endogenous and exogenous sources [8]. UDCA cannot, therefore, be recommended for the purpose of reducing this biochemical marker of disease [8].

Women and pregnant people with ICP do not have increased rates of assisted or operative birth compared with women without ICP [8,11]. Mode of birth should therefore be based on usual obstetric or medical indications [8]. Early delivery (around 35-36 weeks) is indicated in cases of severe ICP (bile acid levels >100 μmol/L). If planned early birth is indicated, induction of labour should be considered unless other reasons for caesarean birth are present [8].

## Conclusions

This case emphasizes the importance for obstetricians to recognize ICP early and institute close feto-maternal monitoring and robust multi-disciplinary care in cases in which pregnant women present with features suggestive of early onset of ICP, especially in cases in which levels of serum bile acids increase rapidly. The presentation of ICP can be puzzling as cases can show variable presentations and progression. There are also documented pieces of evidence of serious and life-threatening foetal and neonatal outcomes associated with ICP, such as stillbirth. When bile acid levels increase quickly and progress fast in ICP, prompt and timely investigation and intervention can help alleviate the patient’s clinical symptoms, while also increasing the chances of optimal foetal outcome. This intervention would often include planning for early delivery when indicated. It is important to emphasize that early recognition, close monitoring, and multi-disciplinary care are key to optimising maternal and foetal outcomes.
